# The Global Burden of Atopic Dermatitis in Elderly Populations: Trends, Disparities, and Future Projections

**DOI:** 10.3390/healthcare13070788

**Published:** 2025-04-01

**Authors:** Zizhuo Li, Jiaxu Gu, Tian Tang, Xinyue Huang, You Wu, Yannan Li, Xia Dou, Bo Yu, Chengxin Li, Han Zhang

**Affiliations:** 1 Department of Dermatology, First Medical Center of Chinese PLA General Hospital, Beijing 100853, China; 2 Department of Dermatology, Peking University Shenzhen Hospital, Shenzhen 518036, China; 3 Shenzhen Key Laboratory for Translational Medicine of Dermatology, Biomedical Research Institute, Shenzhen Peking University-The Hong Kong University of Science and Technology Medical Center, Shenzhen 518036, China; 4 School of Population and Public Health, University of British Columbia, Vancouver, BC V6T 1Z4, Canada

**Keywords:** atopic dermatitis, disease burden, elderly, gender disparities

## Abstract

**Background**: Atopic dermatitis (AD) is a common chronic inflammatory skin disease often affecting infants. However, its significance in adult populations is increasingly recognized. Notably, its prevalence and impact among elderly individuals remain poorly understood, highlighting the need for a deeper understanding of its global burden. This study aims to evaluate the prevalence, incidence, and Disability-Adjusted Life Years (DALYs) of AD in individuals aged 60 and older from 1990 to 2021, with projections to 2045. **Methods**: Data from the Global Burden of Disease (GBD) Study were used to analyze trends in the global burden of AD by region and sex. Key metrics were calculated using annual average percentage changes (AAPC). Based on historical trends, projections for 2022–2045 were developed. **Results**: In 2021, the prevalence of AD in the elderly exhibited substantial regional variation, with the highest rates observed in Northern Europe and North America. Although global prevalence slightly declined from 1990 to 2021, females consistently demonstrated a higher burden than males. Projections indicate a substantial increase in AD cases by 2045, particularly among elderly females, with the 60–64 age group expected to exceed 4 million cases. The disease burden correlated with Universal Health Coverage (UHC) indices, suggesting healthcare access impacts disease reporting and management. **Conclusions**: The increasing burden of AD, especially in elderly females, highlights the urgent need for targeted healthcare strategies to manage AD in aging populations. Further research is required to address regional and gender disparities in AD care.

## 1. Introduction

Atopic dermatitis (AD) is a chronic, inflammatory skin disease characterized by xerosis, widespread eczematous lesions, and intense pruritus [1]. In the chronic course of AD, patients experience recurrent skin lesions and severe itching, which significantly increases the risk of psychological disorders [2,3]. Compared with other chronic diseases, such as hepatitis or cancer, AD has a comparable or even greater negative impact on patients’ health-related quality of life (HRQoL) [4]. In addition to the psychological burden, adult AD patients often face reduced work performance, impacting their income, and bear significant treatment costs, contributing to a substantial socioeconomic burden [5,6].

The global prevalence of AD has steadily increased over the last few decades, making it the most prevalent dermatological disorder worldwide [7]. It affects up to 20% of children and 2–10% of adults worldwide [8,9]. Although AD often manifests in childhood, with many cases resolving by school age, the prevalence and clinical phenotypes among adults have become increasingly recognized [10]. Standardization in diagnostic criteria and broader use have contributed to a higher rate of reported adult AD cases, addressing previous issues of underdiagnoses [11].

Notably, recent studies indicate that elderly patients with AD exhibit distinct disease manifestations and immunological profiles compared with younger populations [12]. Many age-related changes in the skin and immune system resemble key characteristics of AD, including a weakened skin barrier, innate immune dysregulation, increased Staphylococcus aureus infections, and a shift in adaptive immunity towards a type-2 T helper cell response [13,14]. In elderly individuals, AD may have an even greater impact on quality of life due to the presence of comorbidities that contribute to the overall disease burden [15]. Persistent itching can significantly impair sleep quality, while age-related skin dryness exacerbates pruritus, creating a vicious cycle [15].

Additionally, elderly populations face unique challenges in accessing adequate care, particularly in developing regions [16]. Limited coverage by public health insurance or social security systems contributes to lower diagnostic rates and inadequate treatment among elderly patients. As the world’s population keeps aging [17], it is critical to elucidate the epidemiological characteristics of AD in the elderly, which increases awareness and understanding of AD in this age group.

This study aims to analyze global trends in AD among the elderly using Global Burden of Disease (GBD) data from 1990 to 2021. Additionally, this study integrates World Bank economic data to assess the financial burden of AD in elderly populations globally. It also projects the future trajectory of AD burden on the elderly, offering crucial insights for policymakers and healthcare providers to enhance disease management and improve the quality of life for elderly patients with AD.

## 2. Materials and Methods

### 2.1. Overview and Data Collection

The Global Burden of Disease (GBD) study applies a thorough and rigorous framework for measuring global health issues and their effects. It employs a consistent methodology to collect and analyze data from various sources, covering an array of diseases, injuries, and risk factors [18]. The datasets pertaining to atopic dermatitis from the GBD 2021, reflecting the worldwide AD reported by the Global Health Data Exchange, were analyzed. The data were categorized by age, with five-year intervals from 60 to over 95 years, focusing on the global elderly population. Stratification by gender was performed to reflect global demographic trends accurately. The study encompassed 204 countries and territories, providing a comprehensive global perspective. Statistical calculations incorporated 95% confidence intervals to account for inherent data variability, enhancing the reliability and precision of the results. Additionally, data from five newly added SDI regions were included, enriching the dataset and strengthening the analytical capacity to understand health patterns across a broader socioeconomic spectrum [19].

### 2.2. Predict Analysis

This study employed the Bayesian Age-Period-Cohort (BAPC) model, augmented with a random-effects exponential link function, for predictive analytics. Situated within the Bayesian probabilistic framework, the model is optimized for computational efficiency using Integrated Nested Laplace Approximations (INLA), providing an effective alternative to traditional Markov chain Monte Carlo (MCMC) [20] methods. The model’s predictive capacity was utilized to estimate age-standardized prevalence rates (ASPR), age-standardized incidence rates (ASIR), and age-standardized disability-adjusted life year rates (ASDR) for AD over a 23-year period from 2022 to 2045. Additionally, a stratified analysis was conducted, dividing the population into five-year age intervals to allow for a more granular and insightful interpretation of the data.

### 2.3. Health Inequality Analysis

Health inequality is a significant issue in the global public health domain, highlighting the uneven distribution of health outcomes among different socioeconomic groups [21]. This article focuses on the health inequity analysis of elderly atopic dermatitis, drawing attention to the factors leading to unequal access to healthcare services and their subsequent impact on health outcomes. Relative inequality reveals the correlation between health outcomes and socioeconomic status, underscoring the systemic link between health and social standing. Absolute inequality analysis concentrates on the absolute differences in health outcomes, often quantified using the Concentration Index (CI). CI quantifies the distribution of a health variable, such as disease incidence or mortality, across different socioeconomic groups, commonly measuring the degree of inequality in health outcomes based on socioeconomic status. A negative concentration index indicates that health outcomes are more likely to occur in groups with lower socioeconomic status, and the absolute value of the index reflects the severity of the inequality. It is generally considered that an absolute value of CI greater than 0.2 indicates the presence of health inequity. The CI calculation method is as follows: CI *=* $\frac{2}{n}\sum_{i=1}^{n}y_{i}R_{i}-\left(n+1\right)$.

Health inequality analysis enables researchers to identify groups that experience disparities in healthcare access and understand how this affects their health outcomes. This type of analysis assists policymakers and health experts in designing and implementing interventions aimed at reducing health disparities and enhancing the overall health status of communities.

### 2.4. Correlation Analysis

Relevant economic and health metrics, including Current Health Expenditure per capita (in current US dollars), Gross Domestic Product per capita based on Purchasing Power Parity (GDP per capita PPP) (in international US dollars), and the Universal Health Coverage (UHC) service coverage index, were extracted from the World Bank database. Spearman correlation analysis is a non-parametric statistical method used to assess the monotonic relationship between two variables. Unlike the Pearson correlation coefficient, the Spearman correlation coefficient does not require the data to be normally distributed and is less sensitive to outliers, making it particularly suitable for handling non-linear relationships or data with unclear distributions. In this study, we employed Spearman correlation analysis to evaluate the relationship between the ASPR, ASIR, and ASDR of atopic dermatitis (AD) in the elderly and a range of economic and health indicators, such as Current Health Expenditure per capita, Gross Domestic Product per capita based on Purchasing Power Parity (GDP per capita PPP), and the Universal Health Coverage (UHC) service coverage index. To reduce the vast disparities in economic and health indicators across countries, mitigate data skewness, and minimize the impact of outliers, thereby enhancing the robustness and interpretability of the correlation analysis, we applied a log2 transformation to the relevant indicators. This makes the data more comparable and visually interpretable. This analysis aimed to identify potential determinants influencing disease burden, providing strategic insights for disease mitigation and management.

### 2.5. Statistical Analysis

All statistical analyses were performed using the R programming language, version 4.3.3. Statistical significance was defined as a *p* value less than 0.05, obtained from a two-tailed test.

## 3. Results

### 3.1. The Global and Regional Burden of Elderly AD in 2021

**Figure 1:**
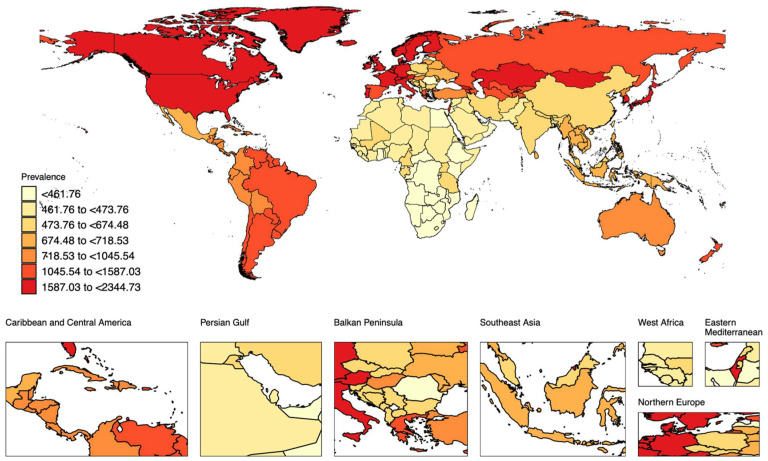
Global distribution of prevalence rates of elderly atopic dermatitis in 2021.

The global prevalence of AD in 2021 was 1017.00 per 100,000 population, which exhibited significant regional variation (Figure 1). Prevalence rates ranged from less than 461.76 to as high as 2344.73 per 100,000 population. The highest prevalence was observed in Northern Europe, North America, and parts of South America, where rates exceeded 1587.03 per 100,000. Moderate prevalence rates, ranging from 718.53 to 1587.03 per 100,000, were reported in Eastern Europe, the Balkan Peninsula, Australia, and parts of the Middle East, including the Persian Gulf. In contrast, the lowest prevalence rates (<461.76 per 100,000) were predominantly found in sub-Saharan Africa, Southeast Asia, and West Africa.

There were also notable regional differences in the Disability-Adjusted Life Years (DALYs) rate of AD in the elderly (Figure S1a). The highest DALY burden, exceeding 63.58 per 100,000 population, was recorded in Northern Europe, parts of North America, and the Balkan Peninsula. Intermediate DALY rates, ranging from 28.82 to 63.58 per 100,000, were observed in Eastern Europe, the Persian Gulf, Australia, and certain regions of the Caribbean and Central America. The lowest DALY rates, under 18.93 per 100,000, were primarily reported in sub-Saharan Africa, Southeast Asia, and West Africa.

Similarly, the incidence rates of AD in 2021 showed considerable geographical variation (Figure S1b). The highest rates, exceeding 190.06 per 100,000 population, were found in Northern Europe, parts of North America, and the Balkan Peninsula. Moderate rates, ranging from 106.20 to 190.06 per 100,000, were observed in Eastern Europe, the Persian Gulf, and selected regions of the Caribbean and Central America. The lowest incidence rates, below 69.33 per 100,000, were largely confined to sub-Saharan Africa, Southeast Asia, and West Africa.

### 3.2. Trends in the Disease Burden in Elderly AD from 1990 to 2021

From 1990 to 2021, the global prevalence of elderly AD decreased from 1117.11 cases per 100,000 (95% UI: 1005.84–1234.26) to 1017.00 cases per 100,000 (95% UI: 915.91–1124.40), reflecting an Average Annual Percent Change (AAPC) of −0.3% (95% CI: −0.32 to −0.28) (Table 1). Analysis by sex revealed a higher prevalence in females, which declined from 1229.35 cases per 100,000 (95% UI: 1108.02–1357.44) in 1990 to 1099.24 cases per 100,000 (95% UI: 991.32–1216.65) in 2021, with an AAPC of −0.36% (95% CI: −0.38 to −0.33). For males, the prevalence decreased from 979.51 cases per 100,000 (95% UI: 879.88–1082.78) in 1990 to 924.82 cases per 100,000 (95% UI: 831.96–1023.50) in 2021, with a smaller AAPC of −0.19% (95% CI: −0.20 to −0.17).

Regarding incidence, the global rate declined from 136.94 per 100,000 (95% UI: 115.24–160.86) in 1990 to 128.22 per 100,000 (95% UI: 108.19–150.09) in 2021, representing an AAPC of −0.21% (95% CI: −0.22 to −0.20). The incidence among females decreased from 145.58 per 100,000 (95% UI: 122.46–171.46) to 134.86 per 100,000 (95% UI: 113.46–158.24), with an AAPC of −0.25% (95% CI: −0.25 to −0.24). Among males, the incidence showed a smaller decrease with an AAPC of −0.15% (95% CI: −0.16 to −0.14).

The global burden of AD, as measured by DALYs, was 171.56 per 100,000 population (95% UI: 150.24–195.65) in 1990 and decreased to 152.44 per 100,000 (95% UI: 132.87–174.05) by 2021, showing an AAPC of −0.35% (95% CI: −0.36 to −0.34). Stratified by sex, females consistently exhibited a higher burden than males over the period. In 1990, DALYs for females were 185.22 (95% UI: 162.35–211.45), compared with 156.34 (95% UI: 137.84–177.82) for males. By 2021, these figures decreased to 164.45 (95% UI: 145.02–186.53) for females and 142.15 (95% UI: 124.67–162.34) for males.

### 3.3. Prediction of the Disease Burden in Elderly AD from 2022 to 2045

**Figure 2:**
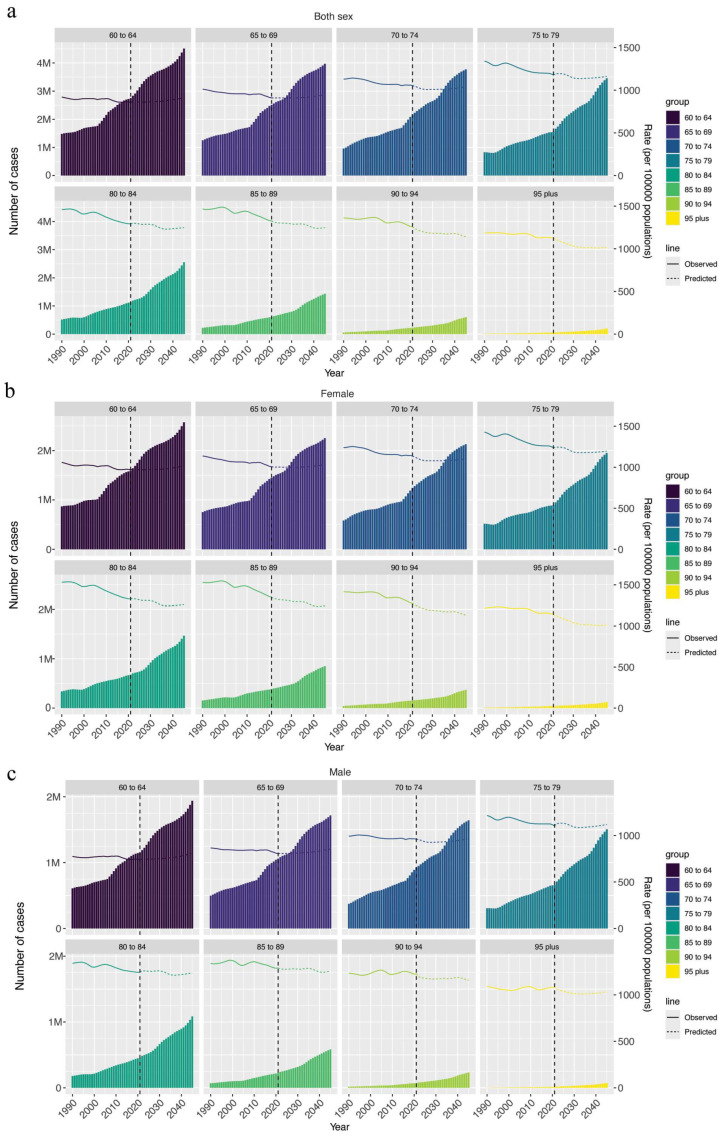
Age-standardized prevalence rate and number of cases in elderly atopic dermatitis by sex, 1990–2040. (**a**). Prevalence rate of elderly atopic dermatitis in both sexes. (**b**). Prevalence rate of elderly atopic dermatitis in females. (**c**). Prevalence rate of elderly atopic dermatitis in males.

The predicted trends in the prevalence, incidence, and DALYs associated with AD among individuals aged 60 and above in various age cohorts indicate a slight decrease in prevalence rates over the coming decades. However, the total number of cases is expected to rise substantially due to population aging. From 2022 to 2045, all age groups are projected to experience a gradual decline in prevalence, but the total case numbers will significantly increase. For instance, the 60–64 age group is expected to exceed 4 million cases by 2045, with the 65–69 and 70–74 age groups projected to surpass 3 million and 2 million cases, respectively (Figure 2a). This upward trend in total case numbers is also evident in older age groups, such as those aged 80–84 and 85–89, underscoring the increasing disease burden within the aging population.

The incidence of elderly AD shows a declining trend across all age groups despite the rising total number of cases (Figure S2a). By 2045, the 60–64 age group is projected to add over 600,000 new cases annually, with similar trends observed in the 65–69 and 70–74 age groups, each exceeding 500,000 new cases. DALYs also demonstrate a declining rate but a substantial increase in the total burden (Figure S2b). Significant increases are also observed in older age groups, such as those aged 80–84 and 85–89, reflecting the growing disease burden within the aging population.

Notably, females consistently account for a higher number of cases compared with males in all age groups (Figure 2). For instance, by 2045, the number of female AD patients aged 60–64 is projected to exceed 2 million cases. Similar gender disparities are observed in older age groups, such as 80–84 and 85–89, where the burden of AD is more pronounced among females.

### 3.4. Health Inequality and Gender Gap in Elderly AD Patients

**Figure 3:**
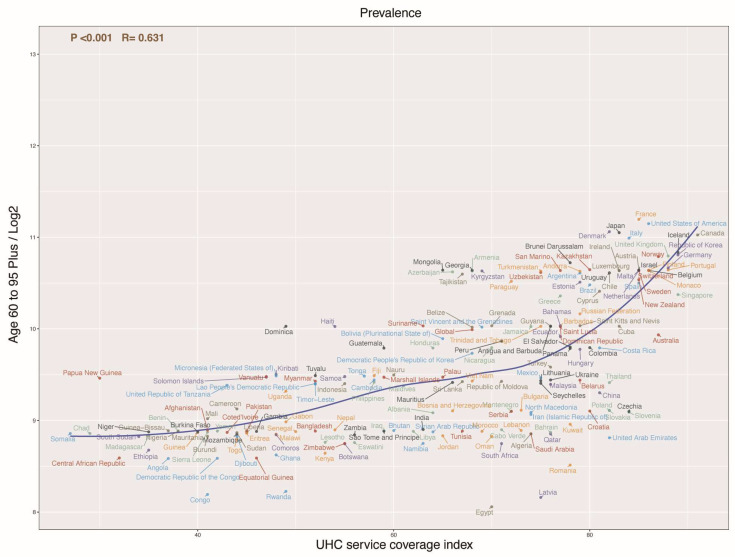
Relationship between Universal Health Coverage (UHC) service coverage index and prevalence of atopic dermatitis (AD) in elderly across various countries. A positive correlation is observed, indicating that higher UHC coverage is associated with a higher reported prevalence of AD.

A significant positive correlation (r = 0.631, *p* < 0.001) was found between the prevalence of AD in individuals aged above 60 years and the Universal Health Coverage (UHC) service coverage index across various countries (Figure 3). This suggests that higher UHC service coverage is associated with increased AD prevalence in the elderly. Countries with high UHC coverage, such as Japan, the United States, and several European nations (e.g., France and Italy), which have coverage indices exceeding 80, reported higher AD prevalence. Conversely, countries with lower UHC coverage, such as those in sub-Saharan Africa (e.g., Chad, Niger, Democratic Republic of the Congo) and parts of Southeast Asia, had lower AD prevalence rates.

**Figure 4:**
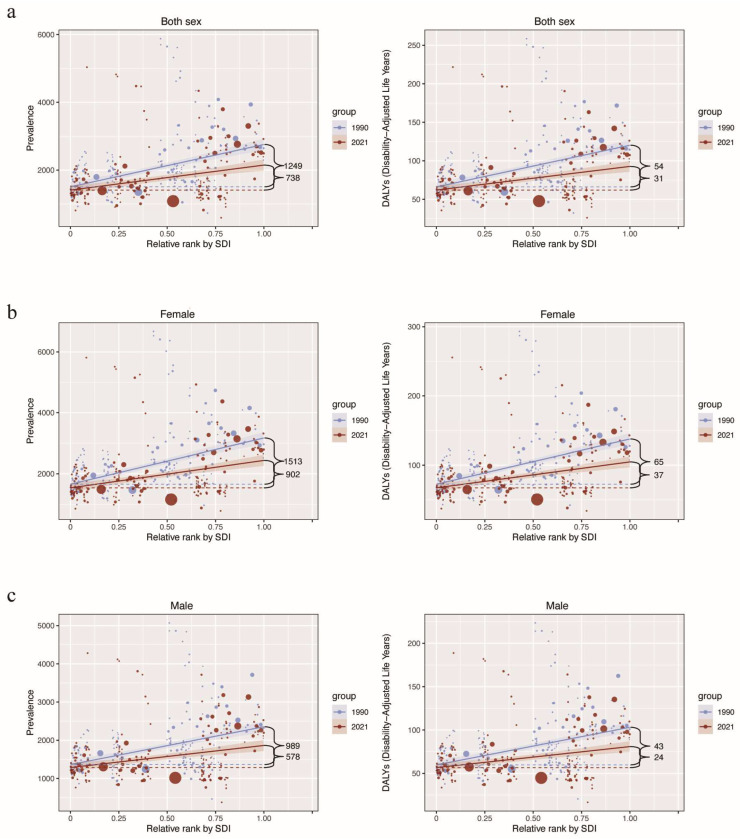
Trends in health inequality among elderly patients with atopic dermatitis (AD) by Social Development Index (SDI) in 1990 and 2021. (**a**) both sexes; (**b**) female; (**c**) male.

In 1990, significant health inequalities were observed in the relationship between prevalence and DALYs of AD and SDI levels among elderly AD patients, with steeper gradients indicating pronounced disparities (Figure 4a). By 2021, these inequalities had moderated, reflecting a reduction in health disparities over time (Figure 4a). However, substantial differences persist, particularly in regions with lower SDI rankings, underscoring the continuing challenge of equitable healthcare delivery for elderly AD patients.

The gender-specific analysis further highlights the disparities. Male elderly AD patients exhibited relatively lower health inequalities than females across all SDI regions (Figure 4b,c). Despite notable improvements in health equity among elderly female AD patients between 1990 and 2021, the inequality coefficient for females in 2021 remained comparable to that of males in 1990, suggesting that progress for females has been slower and disparities remain pronounced (Figure 4b,c).

## 4. Discussion

AD is among the most prevalent inflammatory skin diseases, with a higher occurrence in children and economically developed regions [7,8,9]. Over the past two decades, the global prevalence and incidence of pediatric AD have risen by approximately 5.7 million and 0.7 million cases. However, between 1990 and 2019, the global age-standardized prevalence and incidence rates declined marginally by 0.17% and 0.12%. Regionally, the most significant increases in the number of pediatric AD cases were observed in low-SDI regions, while the largest decreases in age-standardized prevalence and incidence rates occurred in high-SDI regions [22].

In recent years, the growing aging population has brought increased attention to AD in elderly patients [12,15]. While elderly patients with AD often exhibit more localized lesions compared with younger individuals, the impact on sleep and the risk of psychological comorbidities remain comparable across age groups [23]. Age-related changes in skin and immune function further increase the susceptibility of older adults to AD [12]. Additionally, although treatments like dupilumab and tralokinumab have shown promise in managing geriatric AD, many other therapies are contraindicated or poorly tolerated in this population, leading to frequent undertreatment [24,25,26]. Consequently, further research into the management and underlying mechanisms of elderly AD is urgently needed.

This study offers a comprehensive analysis of the global and regional burden of atopic AD in elderly populations, examining trends from 1990 to 2021 and providing projections through 2045. Between 1990 and 2021, the global prevalence and incidence of AD in elderly populations declined by 0.3% and 0.21%, respectively, as shown in Table 1. However, medium–low SDI regions exhibited the most significant increase in prevalence, suggesting that population growth may be a primary factor driving the rise in AD cases across both pediatric and elderly populations. Projections for 2022 to 2045 indicate an anticipated increase in AD prevalence and incidence among elderly populations, particularly within the 60–69 age group, as shown in Figure 2a. These findings underscore the urgent need for a deeper understanding of AD in the elderly.

The global prevalence of AD in the elderly exhibited considerable regional variation. In economically underdeveloped areas—regions where the prevalence, incidence, and DALYs of AD continue to rise, standardized diagnosis and treatment remain less accessible. Historically, diagnostic criteria for atopic dermatitis (AD) have largely been developed and widely adopted by Western countries. However, there are notable differences in the clinical manifestations of AD across different ethnic groups, with variations in features such as flexural involvement, erythema, and lichenification [27]. These ethnic differences are also reflected in the immune subtypes of AD [28]. As a result, the application of these Western-based diagnostic standards may lead to lower detection rates of AD in non-Western regions. In response to these challenges, many developing countries have begun to adapt diagnostic criteria to better reflect the clinical presentation of AD in their populations [29]. With the refinement of diagnostic standards, the detection rate and, consequently, the prevalence of AD in these regions are expected to increase.

Our study also observed a significantly higher proportion of female patients among the elderly, who experience more severe health inequities. A study in China noted that elderly AD patients had a higher male/female ratio and rural/urban ratio than other age groups [30]. However, on a global scale, the number of elderly female patients with AD is significantly greater than that of males, suggesting that older men might have better healthcare access and are more likely to receive treatment. In high-SDI regions, women generally have more resources to focus on their health [31], leading to a higher willingness to seek medical care, whereas in low-SDI regions, women may face greater economic pressures and limited resources, which can hinder their ability to access healthcare. As a result, while the global prevalence of female AD patients is significantly higher than that of males, in developing countries, the male visit rate is often higher. Therefore, addressing gender disparities and health inequities remains crucial in improving healthcare access and treatment outcomes globally.

The impact of environmental and lifestyle factors on AD is crucial in understanding regional trends. Climate change, air pollution, and exposure to allergens such as pollen, dust, and mold can significantly influence AD prevalence and severity. Environmental factors have been associated with increased AD flare-ups [32]. Additionally, lifestyle factors, including diet, hygiene practices, and urbanization, also play a role in the regional variability of AD. As environmental and lifestyle conditions continue to evolve globally, their influence on the burden of AD will likely increase, highlighting the need for region-specific strategies in disease prevention and management. Additionally, genetic and environmental exposures, including air pollution, may have a stronger effect on females, potentially increasing the incidence of AD in elderly women [33].

Healthcare coverage tends to be lower among older adults than younger populations, especially in economically disadvantaged regions. Healthcare insurance is sometimes linked to employment or other forms of social engagement [34]. In many societies, older women may participate less in the workforce or social activities than men, resulting in reduced healthcare insurance coverage and lower healthcare utilization [35]. In our study, both current data and projections for 2045 show that the number of elderly female AD patients surpasses that of elderly male patients. Elderly women face more pronounced health inequalities and remain at higher risk of inadequate disease management. Accordingly, dermatologists should raise awareness of AD in the elderly, especially among older women, and provide more accessible and effective treatment strategies.

As populations continue to age, whether due to early-life disease recurrence or adult-onset disease, the overall number of elderly AD patients will increase. Older adults often face disadvantages in accessing social healthcare resources, comprehensive treatment, and optimal disease management. Elderly women are at even greater risk, as they tend to experience higher levels of pruritus associated with AD [36], further exacerbating their quality of life. The challenges in managing AD in this population are compounded by the presence of multiple comorbidities, which not only complicate treatment regimens but also limit the therapeutic options due to contraindications. Furthermore, elderly individuals often face financial constraints or inadequate healthcare coverage, limiting their access to advanced treatment modalities. The substantial burden of treating AD in the elderly can lead to suboptimal disease management, amplifying both the physical and emotional toll of the condition. Therefore, understanding AD epidemiology in older populations, developing more accurate diagnostic criteria for the elderly, and developing age-specific treatment have significant implications for improving care and outcomes in this vulnerable patient group.

However, several limitations should be acknowledged in interpreting these findings. The reliance on existing epidemiological data may have underestimated AD prevalence in regions with limited medical resources. Additionally, while our projections strongly suggest rising AD cases in the elderly, the underlying pathogenic mechanisms, including immune senescence, lifestyle influences, and genetic predisposition, remain poorly understood. Understanding these factors is crucial for developing targeted interventions to reduce the growing impact of AD in older populations.

Furthermore, the COVID-19 pandemic may have introduced additional biases and complexities in the data. The disruption in healthcare access, increased stress, and changes in lifestyle during the pandemic likely contributed to the exacerbation of AD symptoms, affecting both clinical outcomes and the reported disease burden [37]. The reliance on telemedicine, combined with reduced access to in-person consultations, could have further influenced the quality and consistency of the data collected, particularly for vulnerable populations [38]. These factors should be considered when evaluating the overall impact of AD in 2021.

Additionally, it is important to acknowledge the variability in data quality and healthcare infrastructure across different regions, especially in low-resource settings. Limited access to healthcare services, variations in diagnostic capabilities, and regional differences in healthcare priorities can lead to under-reporting or inaccuracies in AD prevalence and burden data. These gaps are particularly evident in low-income countries, where healthcare systems may be less equipped to provide accurate and consistent data. Such disparities should be considered when interpreting global prevalence estimates and disease burden, as they may contribute to the underrepresentation of AD cases in these regions.

## 5. Conclusions

As the global population ages, the management of elderly health and the long-term control of chronic diseases are becoming increasingly important. AD is one of the most common chronic inflammatory skin conditions, yet research on its prevalence, incidence, and disease burden in the elderly is currently limited. Our study, for the first time, describes the global prevalence, incidence, and DALYs of AD in the elderly, using data from the Global Burden of Disease Study and the World Bank database. We found that in high-SDI regions, AD incidence has slowly declined since 1990, while in low-SDI regions, the incidence continues to rise. This increase may be attributed to the recent implementation of standardized diagnostic criteria and higher healthcare-seeking behaviors among patients in these regions. Despite the declining incidence, the total number of AD cases is still rising due to the growing elderly population. Projections indicate that by 2045, the number of elderly individuals with AD worldwide could reach tens of millions, with a larger proportion of women affected. Furthermore, gender disparities are evident in health inequalities, with elderly women facing more severe challenges in access to healthcare resources and economic burdens. These findings highlight the urgent need for improved diagnosis and management strategies for elderly AD patients, the development of more accurate diagnostic tools for this population, and addressing the healthcare inequalities faced by elderly women. These issues require urgent attention from health policymakers, physicians, and researchers.

## Figures and Tables

**Table 1:** Prevalence, incidence, and DALY trends of atopic dermatitis in the elderly from 1990 to 2021.

	Rate (per 100,000) (95% UI)		
	1990	2021	AAPC (%)
Prevalence			
Global	1117.11 (1005.84–1234.26)	1017.00 (915.91–1124.40)	−0.3 (0.28 to 0.32)
Sex			
Female	1229.35 (1108.02–1357.44)	1099.24 (991.32–1216.65)	−0.36 (−0.38 to −0.33)
Male	979.51 (879.88–1082.78)	924.82 (831.96–1023.50)	−0.19 (−0.20 to −0.17)
SDI regions			
Low SDI	482.46 (427.16–541.41)	483.57 (428.53–542.78)	0.01 (0.01 to 0.02)
Low-middle SDI	578.59 (518.86–643.57)	586.51 (525.58–651.69)	0.04 (0.03 to 0.05)
Middle SDI	697.89 (627.06–772.39)	693.08 (624.80–767.56)	−0.03 (−0.03 to −0.02)
High-middle SDI	1010.72 (905.53–1124.15)	921.59 (824.87–1024.74)	−0.30 (−0.33 to −0.26)
High SDI	1865.91 (1681.75–2059.43)	1839.83 (1657.87–2030.61)	−0.04 (−0.05 to −0.04)
Incidence			
Global	136.94 (115.24–160.86)	128.22 (108.19–150.09)	−0.21 (−0.22 to 0.20)
Sex			
Female	145.58 (122.46–171.46)	134.86 (113.46–158.24)	−0.25 (−0.25 to −0.24)
Male	126.53 (106.73–148.27)	120.78 (101.91–141.52)	−0.15 (−0.16 to −0.14)
SDI regions			
Low SDI	72.34 (60.12–86.03)	72.70 (60.33–86.18)	0.02 (0.017 to 0.025)
Low-middle SDI	84.37 (71.35–98.80)	85.36 (72.06–100.02)	0.034 (0.03 to 0.04)
Middle SDI	103.01 (87.62–119.84)	102.34 (87.59–118.85)	−0.02 (−0.023 to −0.019)
High-middle SDI	130.42 (109.78–153.18)	122.67 (103.49–143.96)	−0.20 (−0.21 to −0.18)
High SDI	205.49 (171.60–243.28)	203.26 (169.80–240.63)	−0.03 (−0.04 to −0.03)
DALY			
Global	44.86 (23.91–75.27)	40.83 (21.77–68.63)	−0.30 (−0.32 to −0.29)
Sex			
Female	49.28 (26.28–82.59)	44.00 (23.49–73.88)	−0.36 (−0.39 to −0.33)
Male	39.47 (21.06–66.46)	37.29 (19.81–62.85)	−0.18 (−0.19 to −0.17)
SDI regions			
Low SDI	19.69 (10.42–34.05)	19.81 (10.45–34.56)	0.03 (0.02–0.04)
Low-middle SDI	23.42 (12.45–40.12)	23.73 (12.63–40.71)	0.04 (0.03–0.06)
Middle SDI	28.48 (15.12–48.61)	28.23 (15.01–48.23)	−0.03 (−0.04 to −0.02)
High-middle SDI	40.67 (21.75–68.40)	37.24 (19.94–63.03)	−0.28 (−0.32 to −0.24)
High SDI	74.66 (39.95–124.69)	73.28 (39.10–122.07)	−0.06 (−0.07 to −0.05)

AAPC, average annual percent change; SDI, Social Development Index; DALY, disability-adjusted life year; UI, uncertainty interval.

## Data Availability

The data presented in this study are available on request from the corresponding author due to institutional data sharing policies.

## Supplementary Materials

The following supporting information can be downloaded at https://www.mdpi.com/article/10.3390/healthcare13070788/s1, Figure S1. Global distribution of incident (a) and DALYs (b) of elderly atopic dermatitis in 2021. Figure S2. Trends and projections of prevalence (a), incident (b), and DALYs (c) in elderly atopic dermatitis from 1990 to 2050.

## Abbreviations

The following abbreviations are used in this manuscript:

AD	Atopic dermatitis
DALYs	Disability-adjusted life years
GBD	Global burden of disease
AAPC	Annual average percentage changes
UHC	Universal health coverage
