# Supplementary material for: The Global Burden of Atopic Dermatitis in Elderly Populations: Trends, Disparities, and Future Projections
Source: Healthcare (Basel). 2025 Apr 1;13(7):788. doi: 10.3390/healthcare13070788 (PMC11988822; doi:10.3390/healthcare13070788)
Supplement: Supplementary file 1 [file healthcare-13-00788-s001.zip › healthcare-3441277-supplementary.pdf]

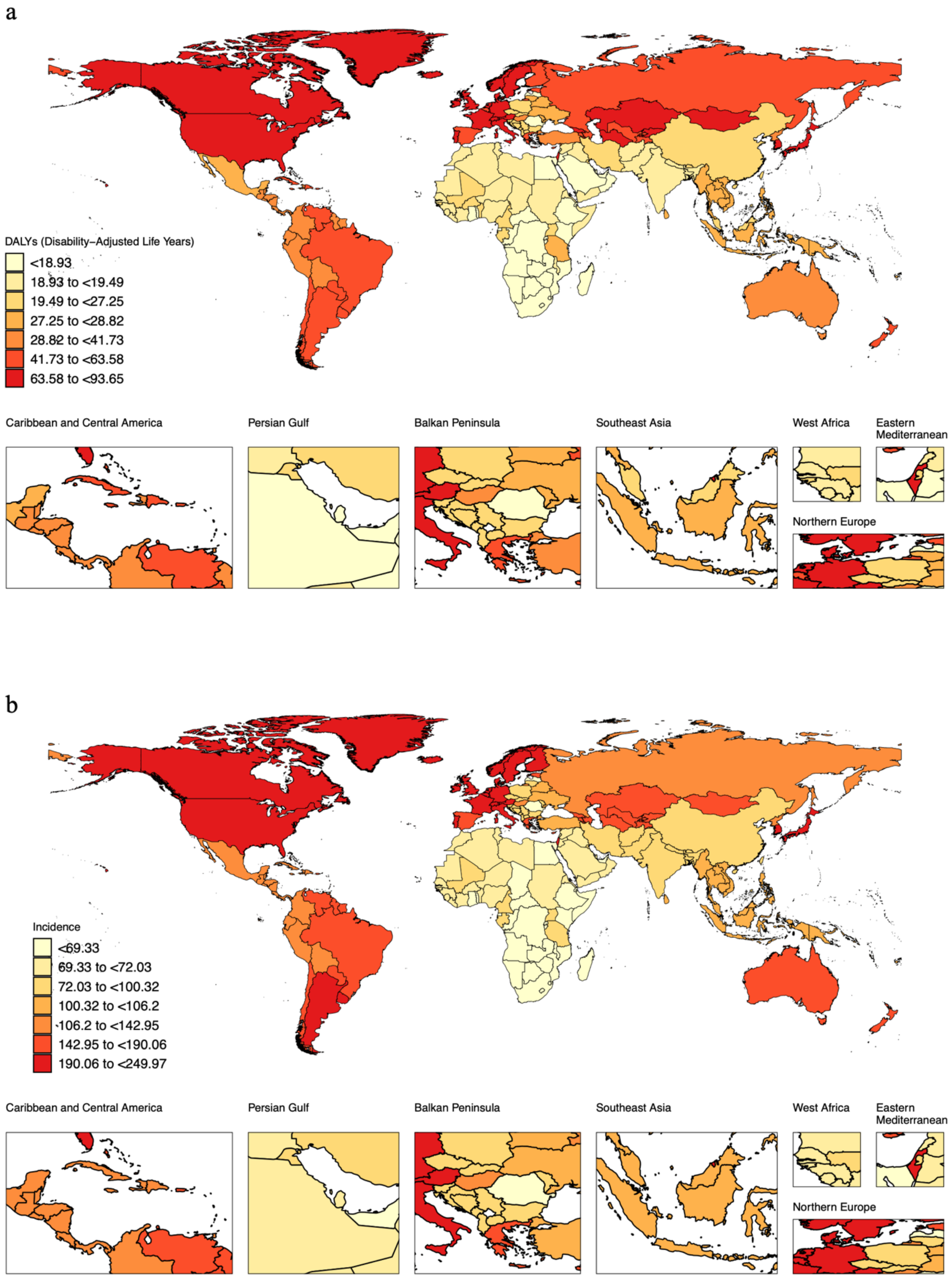

Figure S1. Global distribution of incident (a) and DALYs (b) of elderly atopic dermatitis in 2021.

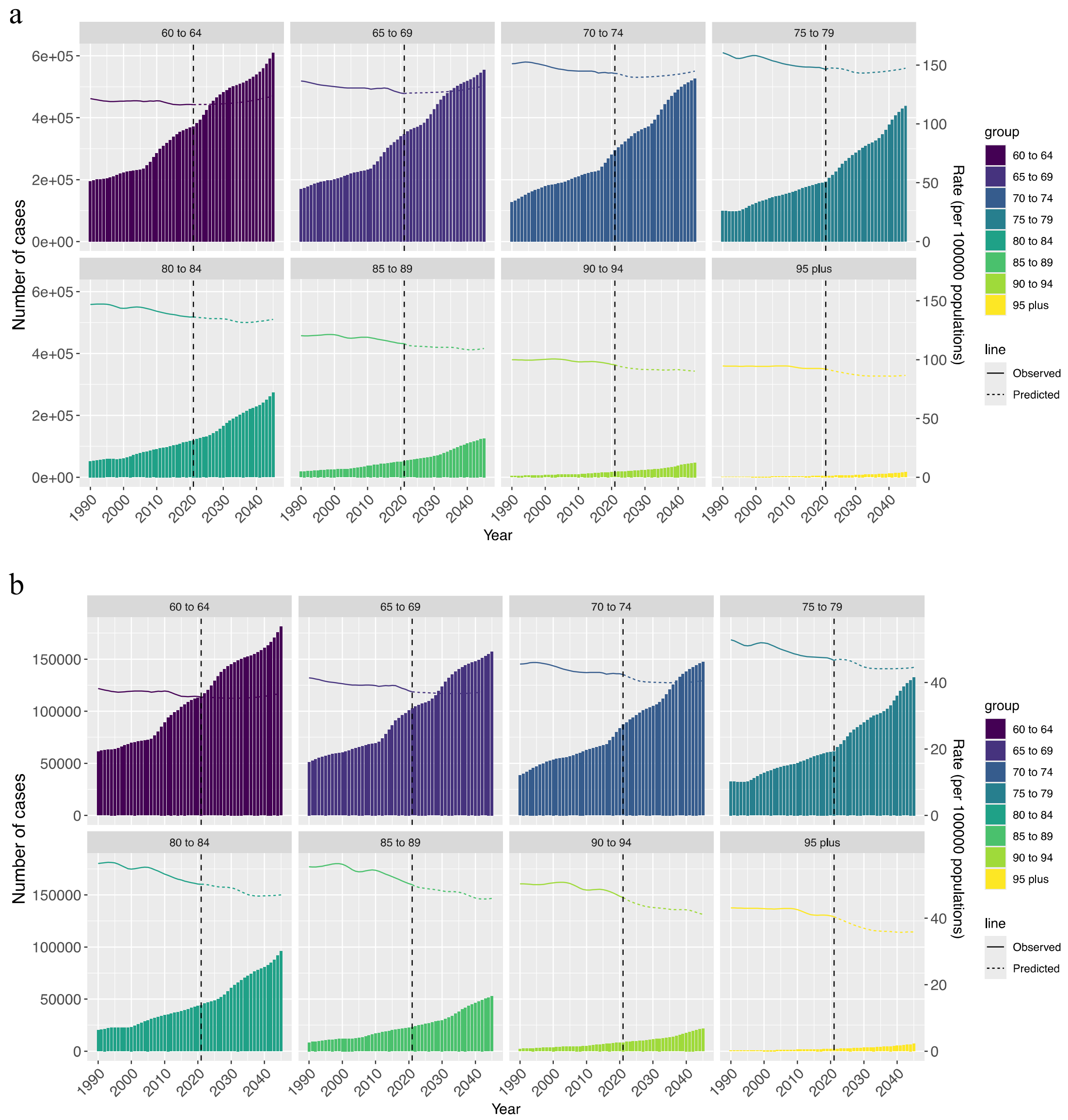

Figure S2. Trends and projections of prevalence (a), incident (b), and DALYs (c) in elderly atopic dermatitis from 1990 to 2050.
